# Engineering of a Microbial Cell Factory for the Extracellular Production of Catalytically Active Phospholipase A_2_ of *Streptomyces violaceoruber*

**DOI:** 10.4014/jmb.2001.01052

**Published:** 2020-06-15

**Authors:** Hyun-Jae Lee†, Ara Cho†, Yeji Hwang, Jin-Byung Park, Sun-Ki Kim

**Affiliations:** 1 Department of Food Science and Technology, Chung-Ang University, Anseong, Gyeonggi 17546, Republic of Korea; 2 Department of Food Science and Engineering, Ewha Womans University, Seoul 03760, Republic of Korea

**Keywords:** A_2_, *Pichia pastoris*, *Escherichia coli*, extracellular production

## Abstract

Phospholipase A_2_ (PLA_2_) from *Streptomyces violaceoruber* is a lipolytic enzyme used in a wide range of industrial applications including production of lysolecithins and enzymatic degumming of edible oils. We have therefore investigated expression and secretion of PLA_2_ in two workhorse microbes, *Pichia pastoris* and *Escherichia coli*. The PLA_2_ was produced to an activity of 0.517 ± 0.012 U/ml in the culture broth of the recombinant *P. pastoris*. On the other hand, recombinant *E. coli* BL21 star (DE3), overexpressing the authentic PLA_2_ (P-PLA_2_), showed activity of 17.0 ± 1.3 U/ml in the intracellular fraction and 21.7 ± 0.7 U/ml in the culture broth. The extracellular PLA_2_ activity obtained with the recombinant *E. coli* system was 3.2-fold higher than the corresponding value reached in a previous study, which employed recombinant *E. coli* BL21 (DE3) overexpressing codon-optimized PLA_2_. Finally, we observed that the extracellular PLA_2_ from the recombinant *E. coli* P-PLA_2_ culture was able to hydrolyze 31.1 g/l of crude soybean lecithin, an industrial substrate, to a conversion yield of approximately 95%. The newly developed *E. coli*-based PLA_2_ expression system led to extracellular production of PLA_2_ to a productivity of 678 U/l•h, corresponding to 157-fold higher than that obtained with the *P. pastoris*-based system. This study will contribute to the extracellular production of a catalytically active PLA_2_.

## Introduction

Phospholipase A_2_ (PLA_2_, EC 3.1.1.4) hydrolyzes the ester bond in the sn-2 position of phospholipids, producing free fatty acids and the corresponding lysophospholipids. In comparison with native lecithins, lysolecithins prepared by PLA_2_ not only exhibit enhanced O/W emulsifying properties but also form stable emulsions under various process conditions [1]. Thus, lysolecithins are used in a wide range of industrial applications such as food, cosmetics, and pharmaceuticals [2]. In particular, PLA_2_ can be used extensively for enzymatic degumming, a key process in the refining of vegetable and other edible oils [3].

Previous studies have been mainly focused on expression and characterization of eukaryotic secretory PLA_2_s. While eukaryotic PLA_2_s have been successfully expressed in yeast [4-7] and fungus [8], inclusion bodies were formed when expressed in *Escherichia coli* due to the presence of five to eight disulfide bonds [9, 10]. Nevertheless, it would be desirable to establish an *E. coli*-based PLA_2_ expression system because eukaryotic systems are generally considered as time consuming and uneconomic in comparison to prokaryotic systems. For this reason, several research groups developed expression systems for soluble expression of the eukaryotic PLA_2_ in *E. coli* using maltose-binding protein (MBP) [11], thioredoxin [12], and protein disulfide bond isomerase (DsbC) [13] as fusion partners. These expression systems, however, require additional steps to eliminate fusion partners and hence are not economic for practical use [14].

It has been relatively easy to express prokaryotic PLA_2_ in *E. coli* because it has only two or zero disulfide bonds [15]. The first PLA identified in prokaryotes was from *Streptomyces violaceoruber* A-2688, a soil bacterium [16]. It is a small protein with molecular weight of 14 kDa containing two disulfide bonds and requires Ca^2+^ for catalytic activity. The PLA_2_ from *S. violaceoruber* was successfully produced extracellularly by *P. pastoris* [17] and *E. coli* [18]. The PLA_2_ expressed in *P. pastoris*, however, contains a part of its signal sequence at the N-terminal end of mature PLA_2_ protein, which might alter properties of the authentic PLA_2_.

In this study, expression and secretion levels of the authentic PLA_2_ in *P. pastoris* and *E. coli* were compared. Since the amount of extracellular PLA_2_ produced in *E. coli* was 8.4 times higher than that in *P. pastoris*, we sought to develop an efficient PLA_2_ expression system in *E. coli*. To do so, effects of the following factors on extracellular production of PLA_2_ were systematically investigated: (1) codon optimization, (2) various host strains, and (3) attachment of aspartate tags.

## Materials and Methods

### Strains and Plasmids

*E. coli* TOP10 strain was used for genetic manipulation, and *P. pastoris* X-33, *E. coli* BL21 star (DE3), Origami 2 (DE3), BL21 (DE3), BL21 RIL (DE3), C41 (DE3), and C43 (DE3) strains were used for PLA_2_ production. For expression of PLA_2_ in *P. pastoris*, codon-optimized PLA_2_ gene was cloned behind the *AOX1* promoter in plasmid pPICZαA and their transcription was induced by adding methanol. Codon optimization was carried out by using the program (https://zendto.bioneer.co.kr/codon/index.py) provided by Bioneer (Korea). For expression of PLA_2_ in *E. coli*, the natural and codon-optimized PLA_2_ genes were located behind the *T7* promoter in plasmid pET- 26b(+) and their transcription was induced by adding isopropyl-β-D-thiogalactopyranoside (IPTG). The natural and codon-optimized PLA_2_ genes were synthesized by Bioneer. Strains and plasmids used in this study are listed in Table 1.

### Genetic Manipulation

The natural PLA_2_ or codon-optimized PLA_2_ genes without native signal sequence (Fig. S1) were PCR amplified with primers of HL01 (with *Msc*I site) and HL02 (with *Xho*I site) or HL03 (with *Msc*I site) and HL04 (with *Xho*I site). After the gene amplification, PCR products were cut with *Msc*I and *Xho*I and then ligated with plasmid pET- 26b(+) digested with the same enzymes to construct pHLK01 and pHLK02 (Table 1). To attach various lengths of aspartate residues at the N-terminal end of PLA_2_, plasmid pHLK01 was amplified with the primer sets and then ligated after *Msc*I treatment. The primer sets used for amplification of the DNA fragments are as follows: HL07 (with *Msc*I site) and HL11 (with *Msc*I site) for pHLK03; HL08 (with *Msc*I site) and HL11 (with *Msc*I site) for pHLK04; HL09 (with *Msc*I site) and HL11 (with *Msc*I site) for pHLK05; HL10 (with *Msc*I site) and HL11 (with *Msc*I site) for pHLK06. Plasmid pSHK01 is identical to pHLK01 except that it does not contain the PelB signal sequence. To make this change, a DNA fragment without PelB signal sequence was amplified with primers SH05 (with *Nde*I site) and SH06 (with *Nde*I site) using pHLK01 as template. This linear DNA was digested with *Nde*I and ligated to construct pSHK01.

**Fig. 1:**
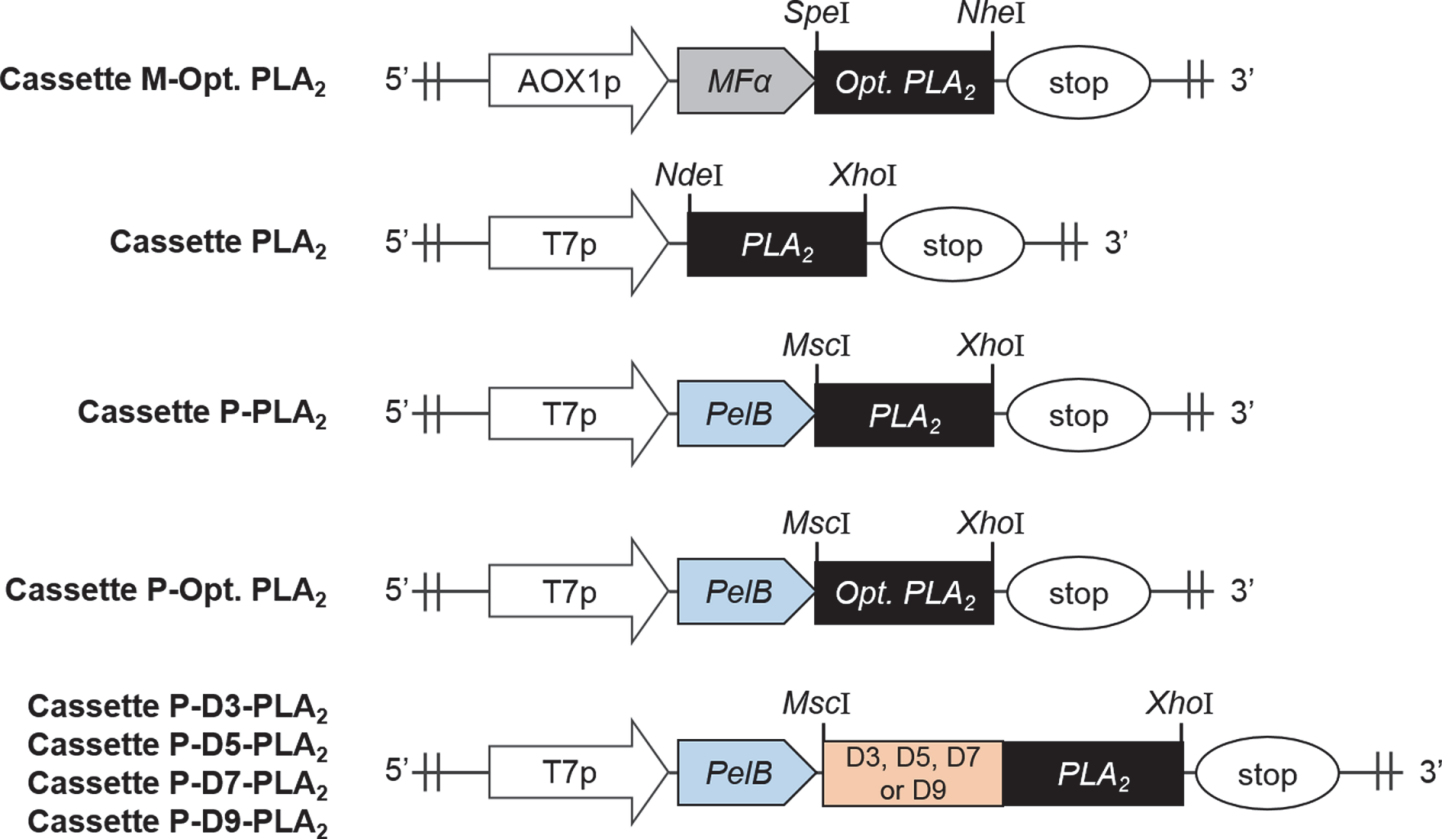
Schematic diagrams of the structures of recombinant PLA_2_ expression cassettes. Symbols: *AOX1* promoter (AOX1p), *T7* promoter (T7p), translational stop codon (stop), and the genes coding for the signal sequence of *S. cerevisiae* mating factor α (*MFα*), the signal sequence of pectate lyase B from *Erwinia carotovora* (*PelB*), *S. violaceoruber* phospholipase A_2_ (*PLA_2_*), codon-optimized PLA_2_ (*Opt. PLA_2_*), 3 aspartates (D3), 5 aspartates (D5), 7 aspartates (D7), and 9 aspartates (D9).

The codon-optimized PLA_2_ gene for expression in *P. pastoris* was PCR-amplified with primers F_PLA_2_ (with *Nhe*I site) and R_PLA_2_ (with *Spe*I site). The plasmid pPICZαA was amplified with primers F_pPICZαA (with *Nhe*I site) and R_pPICZαA (with *Spe*I site). These two linear DNA fragments were digested with *Nhe*I and *Spe*I, and ligated to construct pMFα-PLA_2_. Transformation of the cassette for overexpressing PLA_2_ was performed using the *Pichia* EasyComp Kit (Invitrogen, USA). Plasmid pMFα-PLA_2_ was cut with *Mss*I and transformed. Transformants were selected on YPDS medium (10 g/l yeast extract, 20 g/l peptone, 20 g/l glucose, and 182 g/l sorbitol) containing 100 μg/ml Zeocin. PCR amplification was done with primers (F_ch_AOX1p and R_ch_pPICZα) to verify positive transformants. All plasmids and the check PCR products were sequenced by automatic sequencing (Cosmogenetech, Korea). Names of recombinant PLA_2_ gene products and schematic structures are shown in Fig. 1, and primers used for plasmid constructions and confirmations are listed in Table S1.

### Media and Culture Conditions

*P. pastoris* was pre-cultured in 100 ml BMGY medium (10 g/l yeast extract, 20 g/l peptone, 13.4 g/l yeast nitrogen base, 3 × 10^−4^ g/l biotin, 10 g/l glycerol, and 100 mM potassium phosphate (pH 6.0)) at 30oC and 200 rpm for 24 h. Pre-cultured cells were then inoculated into 100 ml BMMY medium containing 10 g/l yeast extract, 20 g/ l peptone, 13.4 g/l yeast nitrogen base, 3 × 10^−4^ g/l biotin, 5 or 10 g/l methanol, and 100 mM potassium phosphate (pH 6.0). Expression of PLA_2_ was induced by adding methanol every 24 h at a final concentration of 5 or 10 g/l.

*E. coli* cells were pre-cultured in LB medium (5 g/l yeast extract and 10 g/l bacto-trypton) at 37oC and 230 rpm for 12 h. After harvesting the cells, the cell pellets were used for inoculation. Batch fermentations were carried out in a 500 ml baffled flask containing 100 ml of Riesenberg medium [13.5 g/l KH2PO4, 4.0 g/l (NH_4_)_2_HPO_4_, 1.7 g/l citric acid, 1.4 g/l MgSO_4_•7H_2_O, 10 ml/l trace element solution (10 g/l Fe(III) citrate, 2.25 g/l ZnSO_4_•7H_2_O, 1.0 g/l CuSO_4_•5H_2_O, 0.35 g/l MnSO_4_•H_2_O, 0.23 g/l Na_2_B_4_O_7_•10H2O, 0.11 g/l (NH_4_)_6_Mo_7_O_24_, 2.0 g/l CaCl_2_•2H_2_O), pH 6.8] with 20 g/l glucose. Agitation speed was maintained at 200 rpm. When OD_600_ reached 0.8-1.2, 0.2 mM IPTG was added to the culture broth. After induction, cultivation was continued at 25oC for an additional 24 h.

### Preparation of Protein

After IPTG induction of 24 h, the culture broth was centrifuged at 15,000 ×*g* for 10 min to collect the medium fraction. The remaining pellet was resuspended in B-PERTM reagent (Thermo Fisher Scientific, USA) and lysed as specified by the manufacturer. The total, soluble, and insoluble fractions of intracellular proteins were prepared as described in the previous report [19].

### Protein Purification

A 20 ml-scale column containing 750 μl of Ni-NTA agarose (QIAGEN, Germany) was washed with 20 ml of the His-tag binding buffer (pH 7.4) containing 20 mM NaH_2_PO_4_, 20 mM Na_2_HPO_4_, 0.5 M NaCl, and 40 mM imidazole. After 100 ml of the medium fraction prepared as described above was mixed with 300 ml of the His-tag binding buffer, the prepared mixture was loaded into the column. The proteins eluted from the column were collected, and protein concentration was determined using the Bio-Rad protein assay kit with bovine serum albumin (BSA) as the standard.

### Analysis of Protein Expression and Enzyme Assay

To visualize recombinant PLA_2_s, the protein samples were electrophoresed in 12% sodium dodecyl sulfate– polyacrylamide gel, and were either stained using Coomassie blue or were transferred to a polyvinylidene difluoride (PVDF) membrane (Immobilon-P; EMD Millipore, USA). The membrane was then probed with anti- 6X His tag antibody (abcam, UK). After incubating the membrane with goat F(ab')2 Anti-mouse IgG (abcam) conjugated to alkaline phosphatase, the blot was developed using the BCIP/NBT chromogenic substrate solution (SurModics, Eden Prairie, USA) as specified by the manufacturer. The quantification of band intensity was carried out using the densitometry software (Total Lab 1.01; Nonlinear Dynamics Ltd.).

PLA_2_ activity was measured using the sPLA_2_ Assay Kit (Cayman Chemical, USA) according to the manufacturer’s instructions. The absorbance change at 37°C and 414 nm of wavelength was monitored by a spectrophotometer (OPTIZEN POP, Mecasys, Korea) after addition of enzyme solution. One unit (U) of PLA_2_ activity was defined as the amount of PLA_2_ able to hydrolyze 1 μmol of diheptanoyl thio-phosphatidylcholine in one minute.

### Biotransformation of Soybean Lecithin

The 10 ml of reaction mixture was formulated with 0.5 mM Tris-HCl, 6 mM CaCl_2_, 31.1 g/l crude soybean lecithin (Sigma-Aldrich, catalog number P3644) (pH 8.0), and 10% (v/v) of enzyme solution. The reaction conditions of 37°C and 400 rpm were maintained using a stirring heating mantle (LKLAB KOREA, Korea). According to the manufacturer’s information, soybean lecithin consists of an average of 55% (42–63%) L-α- phosphatidylcholine and 20% (10-32%) phosphatidylethanolamine. Concentrations of fatty acids were determined by gas chromatography/mass spectrophotometry (GC/MS), as previously reported [20-22]. Fatty acids present in 500 μl of samples were extracted by mixing with 2 ml of isopropyl alcohol, 500 μl of heptane, and 50 μl of sulfuric acid. For the derivatization, 25 μl of *N*-methyl-*N*-(trimethylsilyl)trifluoroacetamide (TMS) (TCI Chemicals, Tokyo, Japan) dissolved in 75 μl of pyridine was added to 60 μl of sample solution containing lauric acid (TCI Chemicals) as the internal standard. Concentrations of TMS derivatives were determined using GC/MS (Agilent Technologies, USA) equipped with a flame ionization detector, split injection system, and nonpolar capillary column (30 m length, 0.25 mm film thickness, HP-5MS, Agilent Technologies). Column temperature was controlled by the following gradient program: 235°C for 3 min; increase at a rate of 25°C/min; 270°C for 10 min; increase at a rate of 5°C/min; 300°C for 1 min. Mass spectra and scan spectra were obtained by electron impact ionization at 70 eV and within the range of 100–600 m/z, respectively. Selected ion monitoring was used for the detection and fragmentation analysis of the reaction products.

## Results and Discussion

### Production of PLA_2_ in *P. pastoris* X-33

To construct expression plasmid for the PLA_2_ from *S. violaceoruber* in *P. pastoris* X-33, the codon-optimized PLA_2_ gene without its native signal sequence was cloned into pMFα-PLA_2_ vector under the transcriptional control of the alcohol oxidase 1 (*AOX1*) gene promoter [23] containing a signal sequence of *S. cerevisiae* mating factor α (MFα) [24]. A schematic diagram for the PLA_2_ expression cassette in pPICZαA plasmid and its name are displayed in Fig. 1. We noted that recombinant PLA_2_ expressed previously in *P. pastoris* (PLA_2_-Pp) was designed to contain a part of its native signal sequences (Ala-Pro-Pro-Gln-Ala) [17] whereas these five amino acids are not present in the authentic mature PLA_2_ and recombinant PLA_2_ produced in other previous studies (PLA_2_-Ec), which employed *E. coli* as a host strain (Fig. S2) [16, 18, 25]. In addition to the presence of native signal sequences, amino acid sequences of the PLA_2_-Pp were not identical (Fig. S2) to those of the PLA_2_-Ec because these two PLA_2_s were originated from different *S. violaceoruber* sources: the PLA_2_-Pp was from *S. violaceoruber* 2917 whereas the PLA_2_- Ec was from *S. violaceoruber* A-2688. The presence of additional five amino acids at the N-terminal end of PLA_2_- Pp resulted in a lower optimum pH of 6.0 [17] compared to the PLA_2_-Ec, which has optimum pH of 7.3–8.3. In addition to optimum pH, this factor might alter the expression level and some properties of the enzyme as reported for lipase B from *Candida antarctica* (CalB) [26]. Therefore, the PLA_2_-Ec, which does not have its native signal peptide, was used in this study for accurate comparison of PLA_2_ production in *P. pastoris* and *E. coli*.

**Fig. 2:**
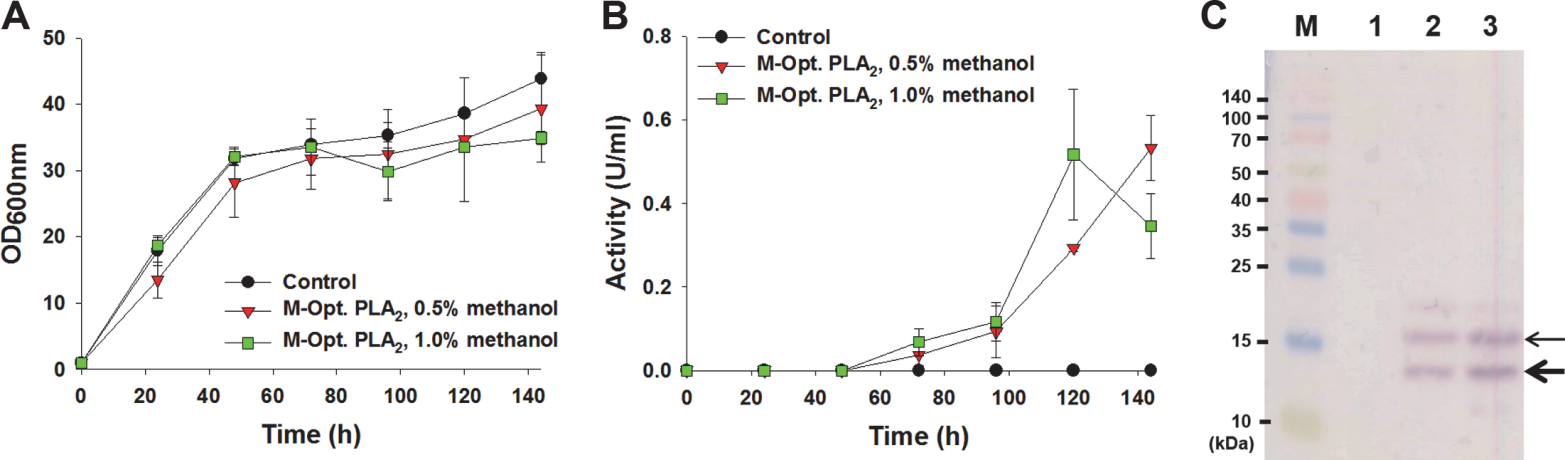
Batch production of PLA_2_ in recombinant *P. pastoris*. (**A** and **B**) growth curves (**A**) and extracellular PLA_2_ activities (**B**) of *P. pastoris* X-33 harboring pPICZαA (Control) and pMFα-PLA_2_. Batch production of PLA_2_ was induced in duplicate by adding 0.5% (v/w) or 1.0% methanol every 24 h. The activities of crude PLA_2_s in the extracellular fraction were measured in triplicate using diheptanoyl thio-phosphatidylcholine as a substrate. (**C**) Western blotting for His-tagged PLA_2_ from the extracellular fraction of the recombinant *P. pastoris strains.* Lanes: M, prestained SDS-PAGE standards; 1, the control strain; 2, *P. pastoris* X-33 harboring pMFα-PLA_2_ induced with 0.5% methanol; 2, *P. pastoris* X-33 harboring pMFα-PLA_2_ induced with 1.0% methanol. The thin and thick arrows point to the protein bands of PLA_2_ with and without glycosylation, respectively.

As expected, growth of the control strain containing the empty plasmid (pPICZαA) and the *P. pastoris* X-33 harboring pMFα-PLA_2_ was virtually identical regardless of methanol concentrations (Fig. 2A), indicating that expression of PLA_2_ in *P. pastoris* had no obvious detrimental effect on growth in general. A batch fermentation of the *P. pastoris* X-33 harboring pMFα-PLA_2_ with intermittent addition of 1.0% methanol led to an extracellular production of PLA_2_-Ec to an activity of 0.517 ± 0.012 U/ml in 120 h (Fig. 2B) (see the Materials and Methods for the activity assay). This value is much lower than the corresponding value (34.7 U/ml) obtained by a batch fermentation of *P. pastoris* overexpressing the PLA_2_-Pp [17]. This is likely due to the difference of PLA_2_ sequences and activity assay methods. While the extracellular PLA_2_ activity with addition of 0.5% methanol was similar to that with 1.0% methanol, addition of 1.0% methanol shortened overall fermentation time from 144 h to 120 h. Although PLA_2_ was difficult to identify using Coomassie blue staining, it was clearly detected by western hybridization analysis using monoclonal anti-His antibodies (Fig. 2C). A band corresponding to the 14 kDa predicted molecular mass of PLA_2_ was visible in both 0.5% and 1.0% methanol induction conditions. A protein band of approximate molecular mass of 16 kDa was also detected (Fig. 2B), and we speculated that this protein band corresponds to glycosylated PLA_2_. This result is consistent with a previous study showing that a part of PLA_2_ expressed in *P. pastoris* was glycosylated as it has three putative glycosylation sites [17].

### Production of PLA_2_ in Various *E. coli* Strains

**Fig. 3:**
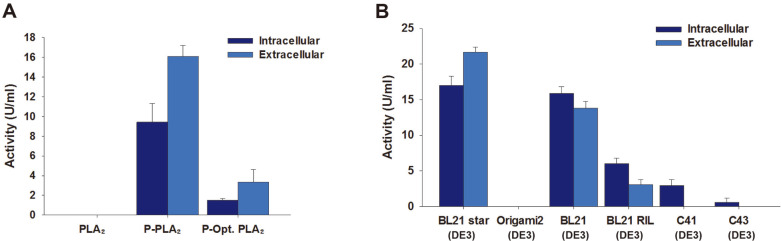
Effects of codon optimization (A) and *E. coli* host strains (B) on activities of recombinant PLA_2_ in intracellular and extracellular fractions. The activities of crude PLA_2_s in the soluble and extracellular fractions (see the Materials and Methods for details) collected 24 h after IPTG induction were measured in triplicate using diheptanoyl thio-phosphatidylcholine as a substrate.

The authentic PLA_2_ gene contains several rare codons for *E. coli* including Leu (CTC). This codon bias problem could be solved by codon optimization of the gene or by supplying rare-codon tRNAs. Here, we investigated effects of codon optimization of PLA_2_ gene on its expression and secretion. The PLA_2_ gene expression system was constructed with and without the PelB signal sequence (Fig. 1), which is involved in targeting the proteins to the periplasmic space [27, 28]. As expected, the PLA_2_ without the signal sequence showed a basal level of lipase activity in both intracellular and extracellular fractions (Fig. 3A). On the other hand, the lipase activities increased up to 9.4 ± 1.5 U/ml in the intracellular fraction and 16.1 ± 0.9 U/ml in the culture broth of the recombinant *E. coli* BL21 star (DE3) overexpressing the authentic PLA_2_ gene (P-PLA_2_). This is 6.3- and 4.8-times higher than the corresponding values of the case of codon-optimized PLA_2_ (P-Opt. PLA_2_) (Fig. 3A). In addition to the enzyme activity assay, SDS–PAGE analysis showed the high secretion of P-PLA_2_ in the culture medium (Fig. S3). This study and earlier studies [29, 30] suggest that a faster expression from the optimized gene could lead to higher concentration of target protein, which in turn results in degradation and/or misfolding of the protein.

Protein expression in *E. coli* BL21 (DE3), BL21 RIL (DE3), BL21 star (DE3), Origami 2 (DE3), C41 (DE3), and C43 (DE3) were analyzed by SDS-PAGE to select a host for PLA_2_ production. The expression level of PLA_2_ was the highest in *E. coli* BL21 star (DE3) which has a mutation in the gene encoding RNaseE (*rne*131 mutation), indicating that protection of mRNAs from RNases plays an important role in PLA_2_ expression (Fig. S4). Therefore, the highest activities in both intracellular and extracellular fractions were obtained for recombinant *E. coli* BL21 star (DE3) overexpressing P-PLA_2_ (Fig. 3B). PLA_2_ activities in culture broth of *E. coli* BL21 (DE3) and BL21 RIL (DE3) exhibited 63.6 and 14.3% of extracellular activity in *E. coli* BL21 star (DE3). Thus, *E. coli* BL21 star (DE3) strain was chosen as the host of PLA_2_ production.

We concluded from these data that alleviating codon bias by codon optimizing the PLA_2_ gene or by supplying rare-codon tRNAs has negative effects on correct folding of PLA_2_. This conclusion supports the hypothesis that the translation is a bottleneck in functional expression of PLA_2_, and hence an overall delay in PLA_2_ expression gives protein machineries more time to fold PLA_2_ correctly.

### Hydrolysis of Soybean Lecithin by Extracellular PLA_2_s from the Recombinant *E. coli* and *P. pastoris*

**Fig. 4:**
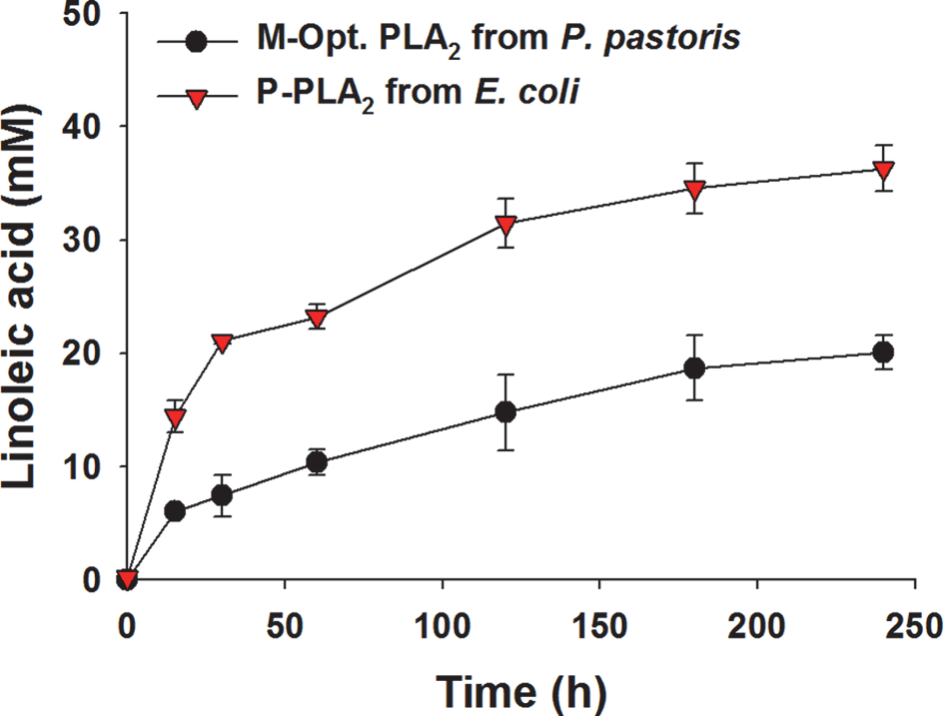
Biotransformation of crude soybean lecithin into linoleic acid and lysolecithin by recombinant PLA_2_ collected from recombinant *P. pastoris* X-33 and *E. coli* BL21 star (DE3) overexpressing PLA_2_. Biotransformation was initiated by adding 10-fold concentrated extracellular crude enzyme solutions from the recombinant *P. pastoris* X-33 and *E. coli* BL21 star (DE3) to the reaction mixture consisting of 0.5 mM Tris-HCl, 6 mM CaCl_2_, and 31.1 g/l crude soybean lecithin (pH 8.0). Results are the mean of triplicate experiments and error bars indicate standard deviations.

The extracellular PLA_2_ activities of the recombinant *E. coli* P-PLA_2_ and *P. pastoris* X-33 M-Opt. PLA_2_ were examined by using an industrial substrate (*i.e*., crude soybean lecithin). When the extracellular fraction of *P. pastoris* X-33 M-Opt. PLA_2_ culture (shown in Fig. 2B) was added into the reaction medium containing 31.1 g/l of crude soybean lecithin (see the Materials and Methods for details), linoleic acid, which was the major fatty acid constituent of soybean lecithin, was produced to 20.1 mM at t = 240 min (Fig. 4). This indicated that approximately 70% of soybean lecithin was hydrolyzed into lysolecithin and linoleic acid. The extracellular fraction of *E. coli* P-PLA_2_ culture displayed a biotransformation profile similar to that of *P. pastoris* X-33 M-Opt. PLA_2_ culture (Fig. 4). Remarkably, linoleic acid was produced to 36.3 mM at t = 240 min (Fig. 4). This indicated that approximately 95% of soybean lecithin was converted into lysolecithin and linoleic acid. Moreover, the initial conversion rate was 2.6-fold greater than that of *P. pastoris* X-33 M-Opt. PLA_2_ culture. Besides, the cultivation time of *E. coli* P-PLA_2_ was significantly shorter than that of *P. pastoris* X-33 M-Opt. PLA_2_ (32 h vs. 120 h). It was thereby assumed that the *E. coli*-based PLA_2_ expression system would be superior to the *P. pastoris* system in terms of extracellular PLA_2_ productivity.

### Effects of N-terminal Repeat of Aspartate Residues on Specific Activity and Expression of PLA_2_

**Fig. 5:**
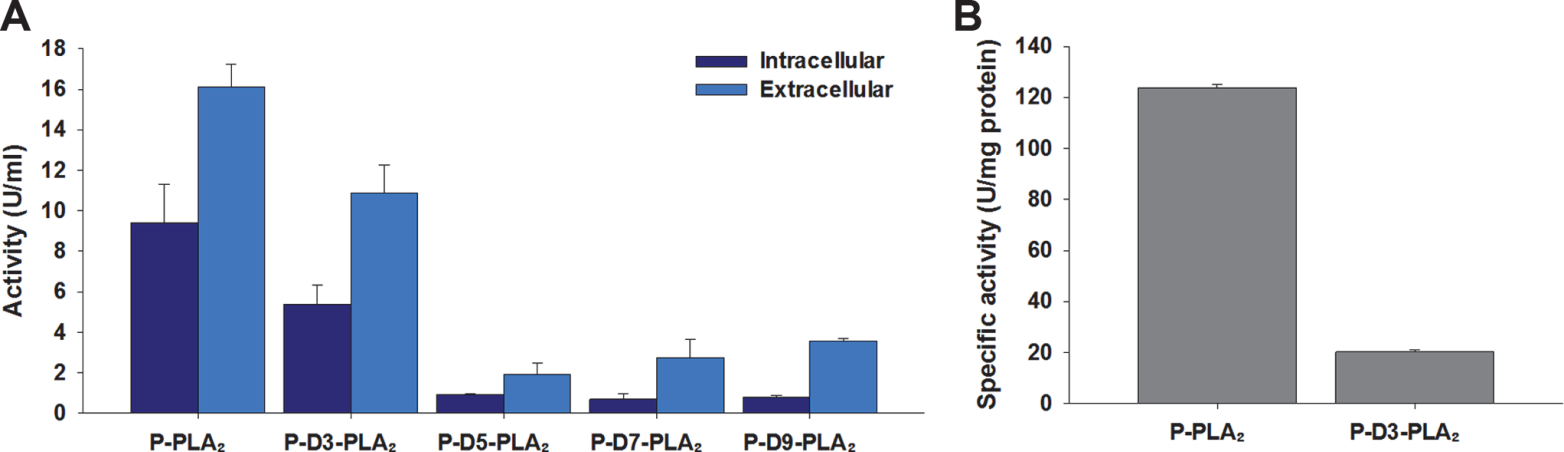
Activity assays of recombinant PLA_2_s with various lengths of aspartate tags (A) and His-tag purified P-PLA_2_ and P-D3-PLA_2_ (B) to investigate the effects of aspartate tags on expression in *E. coli* and specific activity of PLA_2_. Results are the mean of triplicate experiments and error bars indicate standard deviations.

We previously reported that fusion tag systems composed of the PelB signal sequence and repeated aspartate tags improved both expression and secretion of CalB and asparaginase isozyme II (AnsB) from *E. coli* [31, 32]. To investigate whether or not repeated aspartate residues would improve the secretion and activity of PLA_2_, various lengths of aspartate residues were introduced into the N-terminal end of PLA_2_ gene to construct the cassettes P- D3-PLA_2_, P-D5-PLA_2_, P-D7-PLA_2_, and P-D9-PLA_2_ as shown in Fig. 1. Crude PLA_2_ enzymes present in the intracellular and extracellular fractions were subjected to SDS-PAGE (Fig. S5) and activity (Fig. 5A) analyses. Among a series of repeated amino acids consisting of 3, 5, 7, or 9 aspartates, the three aspartates facilitated the secretion of PLA_2_, and hence comparison of the band intensities from the extracellular fractions showed that the band corresponding to the P-D3-PLA_2_ was 64% greater than P-PLA_2_ (Fig. S5). However, the intracellular and extracellular lipase activities obtained for recombinant *E. coli* BL21 star (DE3) overexpressing P-D3-PLA_2_ were instead 42.6% and 32.3% lower than the corresponding values obtained in the case of P-PLA_2_ (Fig. 5A). These results suggested that the presence of three aspartate residues at the N-terminal end of PLA_2_ might alter the specific activity of PLA_2_. To confirm the hypothesis, PLA_2_ and P-D3-PLA_2_ were His-tag purified and subjected to activity assay. As expected, specific activity of P-D3-PLA_2_ was 6.1 times lower than that of PLA_2_ (Fig. 5B). This result is consistent with previous studies reporting that the attachment of repeated aspartates altered catalytic efficiency of CalB and α-1,2-fucosyltransferase (FucT2) from *Helicobacter pylori* [31, 33]. More research is in progress to find PLA_2_ from other bacteria with increased stability, of which specific activity is not affected significantly by the attachment of repeated aspartates.

In conclusion, this study demonstrated that an *E. coli*-based PLA_2_ production system could be more efficient in terms of PLA_2_ productivity, as compared to the *P. pastoris*-based system. Among the *E. coli* host strains harboring the authentic PLA_2_ gene (P-PLA_2_) or codon- optimized PLA_2_ (P-Opt. PLA_2_), the recombinant *E. coli* BL21 star (DE3) P-PLA_2_ has exhibited the highest activities of 21.7 ± 0.7 U/ml in the culture broth and 17.0 ± 1.3 U/ml in the intracellular fraction. Moreover, the extracellular PLA_2_s from the recombinant *E. coli* P-PLA_2_ culture was able to hydrolyze 31.1 g/l of crude soybean lecithin to linoleic acid and lysolecithin at a conversion yield of at least 95%. Therefore, it was concluded that the recombinant *E. coli* P-PLA_2_ system could be used as a microbial cell factory to produce a catalytically active PLA_2_ for hydrolysis of the selective sn-2 position of plant lecithins.

## Supplemental Materials



Supplementary data for this paper are available on-line only at http://jmb.or.kr.

## Figures and Tables

**Table 1:** Strains and plasmids used in this study.

Name	Description	Reference
*E. coli*		
*E. coli* TOP10	F^-^ *mcrA Δ(mrr-hsdRMS-mcrBC) Φ80lacZΔM15 ΔlacX74 recA1 araD139 Δ(ara-leu)7697 galU galK rpsL(Str^R^) endA1 nupG*	Invitrogen
(Carlsbad, CA, USA)
*E. coli* BL21 (DE3)	F^-^ *ompT hsd*S (rB- mB-) *dcm gal* (DE3 [*lac*I *lac*UV5-T7 gene 1 *ind*1 *sam*7 *nin*5])	Novagen
(Darmstadt, Germany)
*E. coli* BL21 star (DE3)	BL21 *rne131* (DE3)	Invitrogen
*E. coli* BL21 CodonPlus-RIL (DE3)	BL21 (DE3) *dcm^+^* Tet^r^ *endA* Hte [*argU ileY leuW* Cam′]	Agilent technologies
(Santa Clara, CA, USA)
*E. coli* C41 (DE3)	BL21 (DE3 *[lacI lac-T7 gene 1 ind1 sam7 nin5]*)	Lucigen
(Middleton, WI, USA)
*E. coli* C43 (DE3)	C41 (DE3) derivative	Lucigen
*E. coli* Origami (DE3)	*∆ara–leu7697 ∆lacX74 ∆phoAPvuII phoR araD139 ahpC galE galK rpsL* F*'[lac^+^(lacI^q^)pro] gor522 ::Tn10 (Tet^R^) trxB::kan* (DE3)	Novagen
SK33	BL21 star (DE3) containing pET-26b(+)	This study
SK31	BL21 star (DE3) containing pHLK01	This study
SK32	BL21 star (DE3) containing pHLK02	This study
SK35	Origami (DE3) containing pHLK01	This study
SK36	BL21 (DE3) containing pHLK01	This study
SK37	BL21 RIL (DE3) containing pHLK01	This study
SK38	C41 (DE3) containing pHLK01	This study
SK39	C43 (DE3) containing pHLK01	This study
SK54	BL21 star (DE3) containing pHLK03	This study
SK55	BL21 star (DE3) containing pHLK04	This study
SK56	BL21 star(DE3) containing pHLK05	This study
SK57	BL21 star(DE3) containing pHLK06	This study
SK87	BL21 star(DE3) containing pSHK01	This study
*P. pastoris*		
*P. pastoris* X-33	Wild type	Invitrogen
PX	X-33 containing pPICZαA	This study
PP	X-33 containing pMFα-PLA_2_	This study
Plasmids		
pET-26b(+)	pBR322 origin, *T7* promoter, PelB signal sequence, His-tag, Kan^R^	Novagen
pHLK01	Expression vector containing P-PLA_2_, Kan^R^	This study
pHLK02	Expression vector containing P-Opt. PLA_2_, Kan^R^	This study
pHLK03	Expression vector containing P-D3-PLA_2_, Kan^R^	This study
pHLK04	Expression vector containing P-D5-PLA_2_, Kan^R^	This study
pHLK05	Expression vector containing P-D7-PLA_2_, Kan^R^	This study
pHLK06	Expression vector containing P-D9-PLA_2_, Kan^R^	This study
pSHK01	Expression vector containing PLA_2_, Kan^R^	This study
pPICZαA	pUC origin, *AOX1* promoter, MFα signal sequence, His-tag, Zeocin^R^	Invitrogen
pMFα-PLA_2_	Expression vector containing M-Opt. PLA_2_, Zeocin^R^	This study
