# Supplementary material for: Engineering of a Microbial Cell Factory for the Extracellular Production of Catalytically Active Phospholipase A2 of Streptomyces violaceoruber
Source: J Microbiol Biotechnol. 2020 Jun 15;30(8):1244–51. doi: 10.4014/jmb.2001.01052 (PMC9728194; doi:10.4014/jmb.2001.01052)
Supplement: Supplementary file 1 [file JMB-30-8-1244-supple.pdf]

J. Microbiol. Biotechnol.
https://doi.org/10.4014/jmb.2001.01052

Supplementary data

Table S1. List of primers used in this study.

Name	Sequence (5' → 3')	Restriction enzyme	Description
HL01	GGCG*TGGCCA*TGGCCCCCGCGGAC	*Msc*I	To construct pHLK01
HL02	CCG*CTCGAG*GCCGAAGATCTTGACGG	*Xho*I	
HL03	GGCG*TGGCCA*TGGCCCCGGCGGATAAAC	*Msc*I	To construct pHLK02
HL04	CCG*CTCGAG*GCCGAAGATCTTCACCGC	*Xho*I	
HL07	GGCG*TGGCCA*TGGATGATGATGCCCCCGCGGAC	*Msc*I	To construct pHLK03
HL11	CCCG*TGGCCA*TCGCCGG	*Msc*I	
HL08	GGCG*TGGCCA*TGGATGATGATGATGATGCCCCCGCGGAC	*Msc*I	To construct pHLK04
HL11	CCCG*TGGCCA*TCGCCGG	*Msc*I	
HL09	GGCG*TGGCCA*TGGATGATGATGATGATGATGATGCCCC CGCGGAC	*Msc*I	To construct pHLK05
HL11	CCCG*TGGCCA*TCGCCGG	*Msc*I	
HL10	GGCG*TGGCCA*TGGATGATGATGATGATGATGATGATGATGCCCCCGCGGAC	*Msc*I	To construct pHLK06
HL11	CCCG*TGGCCA*TCGCCGG	*Msc*I	
SH05	GGAATTC*CATATG*GCCCCCGCGGAC	*Nde*I	To construct pSHK01
SH06	GGAATTC*CATATG*TATATCTCCTTCTTAAAGTTAAACAAAATTATTTCT	*Nde*I	
F_pPICZαA	CTA*GCTAGC*CATCATCATCATCATCATTGAGTTTGT	*Nhe*I	To construct pMFα-PLA2
R_pPICZαA	G*ACTAGT*AGCTTCAGCCTCTCTTTTCTC	*Spe*I	
F_PLA_2	CTA*GCTAGC*TCCAAAAATCTTAACTGCTTGATAATAAGT	*Nhe*I	To construct pMFα-PLA2
R_PLA_2	G*ACTAGT*GCTCCTGCAGACAAACC	*Spe*I	
F_ch_AOX1p	GTTGGTATTGTGAAATAGACGCA	-	To confirm transformants
R_ch_pPICZα	TGAGTTTTTGTTCTAGAAAGCTGG	-	

P-PLA$_2$

ATGAAATACCTGCTGCCGACCGCTGCTGCTGGTCTGCTGCTCCTCGCTGCCCAGCCGGCGATGGCCATGGCCCCCGCGGACAAGCCCCAGGTACTCGCCTC
CTTCACGCAGACCAGCGCGTCCAGCCAGAACGCCTGGCTCGCGGCCAACCGGAACCAGTCCGCCTGGGCCGCCTACGAGTTCGACTGGTCCACGGACCTG
TGCACCCAGGCGCCCGACAACCCCTTCGGCTTCCCGTTCAACACGGCCTGCGCGCGCCACGACTTCGGTTACCGCAACTACAAGGCGGCGGGCAGCTTCG
ACGCCAACAAGAGCCGTATCGACAGCGCCTTCTACGAGGACATGAAGCGCGTCTGCACCGGCTACACCGGCGAGAAGAACACGGCCTGCAACAGCACCG
CCTGGACCTACTACCAGGCCGTCAAGATCTTCGGCCTCGAGCACCACCACCACCACCACTGA

P-Opt. PLA$_2$

ATGAAATACCTGCTGCCGACCGCTGCTGCTGGTCTGCTGCTCCTCGCTGCCCAGCCGGCGATGGCCATGGCCCCGGCGGATAAACCTCAGGTTCTGGCTTC
ATTTACGCAGACCTCGGCGTCCAGTCAAAATGCGTGGCTGGCGGCAAACCGTAATCAGTCGGCATGGGCAGCATATGAATTCGATTGGTCTACAGATCTGTG
TACCCAGGCGCCAGACAACCCGTTCGGCTTTCCGTTTAATACTGCGTGCGCTCGCCATGATTTTGGTTACCGCAACTACAAAGCCGCTGGTAGTTTCGATGC
AAATAAATCTCGTATTGATAGCGCCTTTTATGAGGACATGAAACGTGTGTGTACCGGGTATACGGGTGAAAAAAACACGGCCTGCAATAGCACTGCCTGGAC
CTATTATCAAGCGGTGAAGATCTTCGGCCTCGAGCACCACCACCACCACCACTGA

Supplementary figure 1. DNA sequences of the natural PLA$_2$ (P-PLA$_2$) and codon optimized PLA$_2$ (P-Opt. PLA$_2$). The letters in red, blue, and orange represent PelB signal sequence, PLA$_2$ without native signal sequence, and His-tag, respectively.

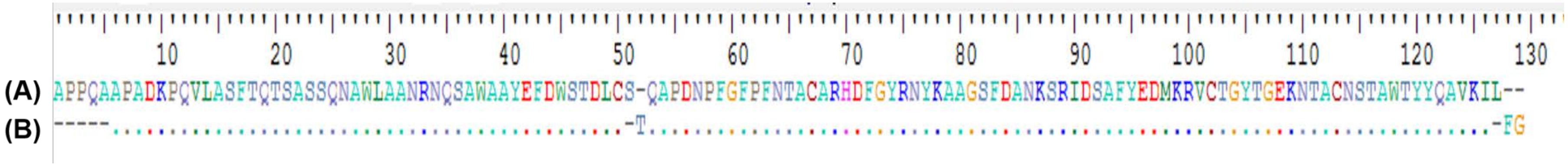

Supplementary figure 2. Amino-acid sequence alignment of PLA$_2$s. The amino-acid sequences from the PLA$_2$-Pp (A) and PLA$_2$-Ec (B) are compared using BioEdit.

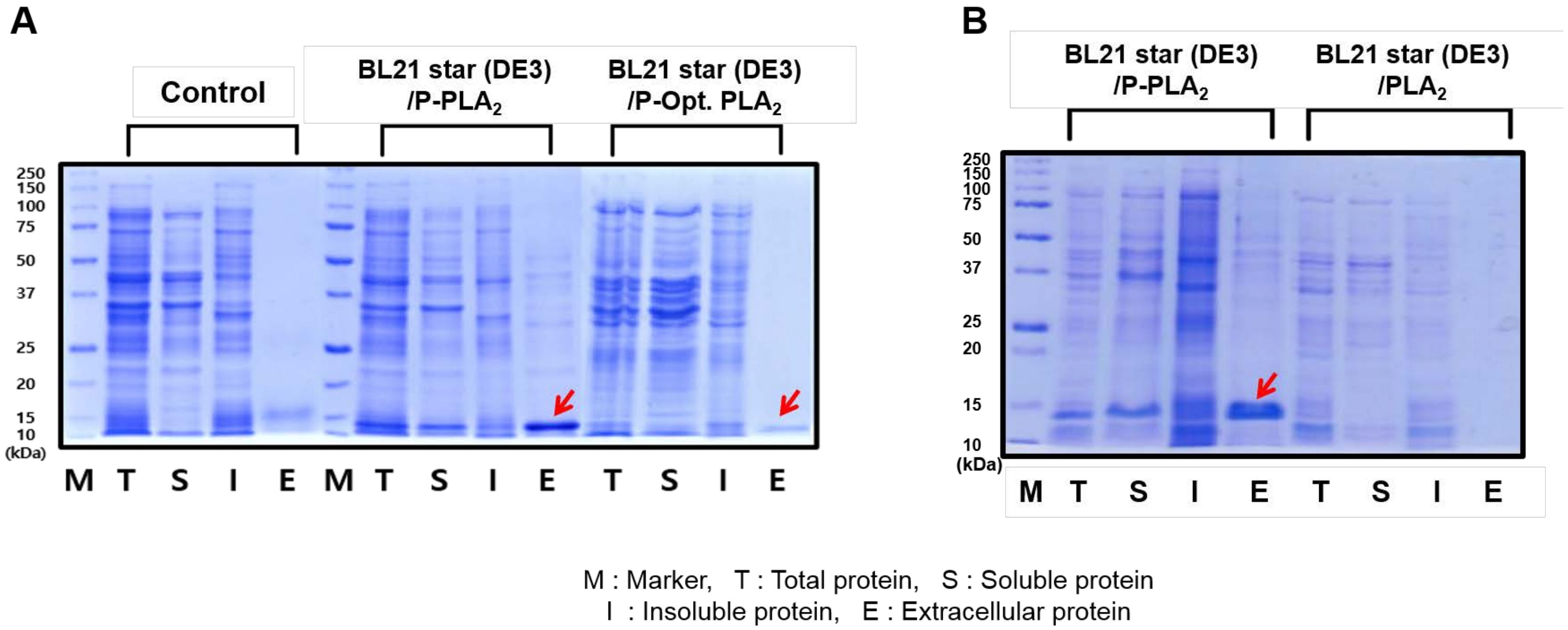

Supplementary Figure 3. SDS-PAGE analysis of recombinant PLA_2s to investigate the effects of codon optimization (A) and the attachment of PelB signal peptide (B) on expression and secretion of PLA_2. After 24 h induction, the cells were harvested, disrupted and fractionated into total (T), soluble (S), insoluble (I) and extracellular (E) protein fractions. P-PLA_2, P-Opt.PLA_2, and PLA_2 represent the natural PLA_2 with PelB signal peptide, codon optimized PLA_2 with PelB signal peptide, and the natural PLA_2 without PelB signal peptide, respectively. The arrow points the protein band of recombinant PLA_2s.

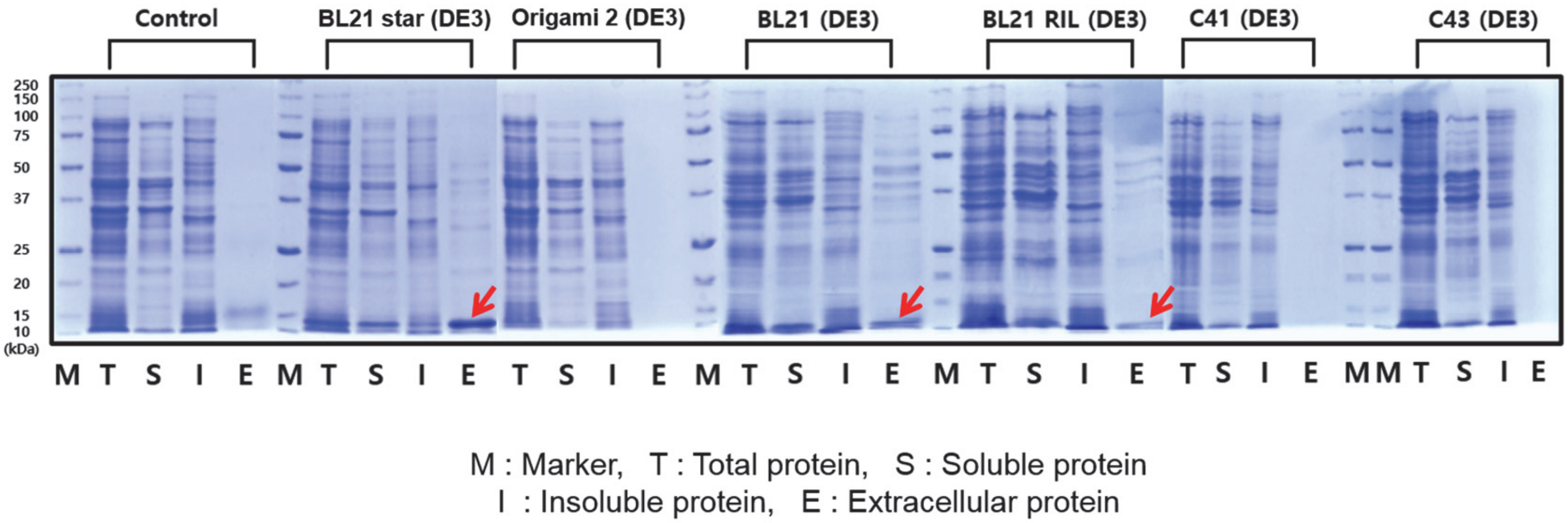

Supplementary Figure 4. SDS-PAGE analysis of recombinant PLA_2 expressed in various *E. coli* host strains. After 24 h induction, the cells were harvested, disrupted and fractionated into total (T), soluble (S), insoluble (I) and extracellular (E) protein fractions. The arrow points the protein band of recombinant PLA_2.

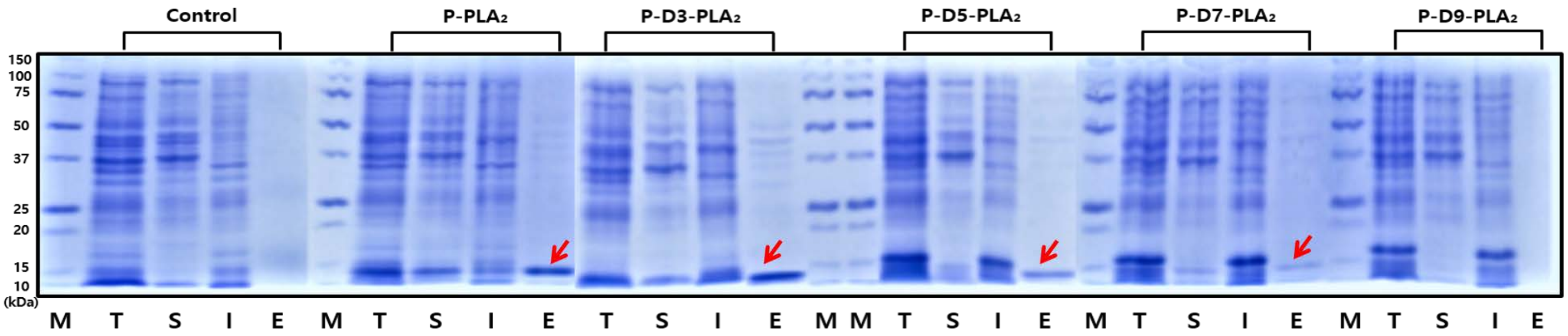

M : Marker, T : Total protein, S : Soluble protein
I : Insoluble protein, E : Extracellular protein

Supplementary Figure 5. SDS-PAGE analysis of recombinant PLA_2s with various lengths of aspartate tags. After 24 h induction, the cells were harvested, disrupted and fractionated into total (T), soluble (S), insoluble (I) and extracellular (E) protein fractions. The arrow points the protein band of recombinant PLA_2.
